# Prevalence, microbiological profiles, and determinants of hospital-acquired pneumonia in Addis Ababa: A focus on *Pseudomonas aeruginosa* and its antimicrobial resistance patterns in three hospitals

**DOI:** 10.1371/journal.pone.0340680

**Published:** 2026-01-20

**Authors:** Etsub Brhanesilassie Hailemichael, Adey Feleke Desta, Girma Taye, Sirak Robele Gari, Etsehiwot Adamu, Zeleke Ayenew, Wondwossen Amogne

**Affiliations:** 1 Ethiopian Water Resources Institute, Addis Ababa University, Addis Ababa, Ethiopia; 2 Africa Centre of Excellence for Water Management, Addis Ababa University, Addis Ababa, Ethiopia; 3 Department of Microbial Sciences and Genetics, Addis Ababa University, Addis Ababa, Ethiopia; 4 College of Health Sciences, Addis Ababa University, Addis Ababa, Ethiopia; 5 CIMA/ARNET-Centre for Marine and Environmental Research/ Aquatic Research Network, Universidade do Algarve, Campus de Gambelas, Faro, Portugal; 6 Ethiopian Public Health Institute, Addis Ababa, Ethiopia; 7 Department of Internal Medicine, Division of Infectious Diseases, School of Medicine, Addis Ababa University, Addis Ababa, Ethiopia; Children's National Hospital, George Washington University, UNITED STATES OF AMERICA

## Abstract

Hospital-acquired pneumonia is typically polymicrobial; nevertheless, *Pseudomonas aeruginosa* is a principal causative pathogen, attributable to its link with poor clinical prognoses and extensive antimicrobial resistance. Our study aims to assess the prevalence, microbiological profiles and determinants of hospital-acquired pneumonia with a focus on antibiotic-resistant *P. aeruginosa* across three hospitals in Addis Ababa. A cross-sectional study was conducted in which 1,800 patients were screened, and 298 cases of hospital-acquired pneumonia were identified between September 2022 and April 2024. Patient interviews and microbiological analysis of lower respiratory tract samples were performed. We detected a 17% prevalence of hospital-acquired pneumonia and 19% prevalence of ventilator-associated pneumonia across the study hospitals. Our patient profiles indicated a predominance of males (59%), with the largest proportion aged 30–39 years (28%), most were married (71%) and had attained secondary-level education (33%). Over half of the patients were admitted to the adult ICU (55%), 60% had a history of prior hospitalization and respiratory disease was the leading cause of admission (30%). *Acinetobacter baumannii* (n = 24) was the most frequently isolated pathogen, followed by *Pseudomonas aeruginosa* (n = 21) and *Staphylococcus aureus* (n = 13). Compounding these challenges, the *P. aeruginosa* isolates (7%) exhibited high resistance to ceftazidime and cefepime (89% resistance), while retaining relatively high susceptibility to amikacin (90%); notably, 67% of the isolates were multidrug resistant. We tested several patient-level vulnerabilities, only aspiration remained independently associated with presence of pneumonia-associated pathogen in patient samples (AOR = 4.43, 95% CI: 1.74–11.24, p = 0.002). This study demonstrates a substantial burden of multidrug resistance hospital-acquired pneumonia by ESKAPE pathogens that indicate deficiencies in hospital defences against hospital-acquired pathogens and risk of adverse patient outcomes. There is an urgent need to shift infection prevention strategies, emphasizing aspiration prevention measures and strengthened diagnostic stewardship.

## Introduction

Hospital-acquired pneumonia and ventilator-associated pneumonia pose substantial challenges to healthcare systems, jointly accounting for roughly 22% of all healthcare-associated infections [1]. They also form part of the most severe healthcare-associated infections, contributing substantially to morbidity, mortality, prolonged hospital stays and increased healthcare costs worldwide [2]. Hospital-acquired pneumonia refers to a pulmonary infection that emerges 48 hours or more after hospital admission while ventilator-associated pneumonia is a form of hospital-acquired pneumonia manifesting 48–72 hours following intubation or placement of an artificial airway [3].

In high-income countries, the estimated incidence of hospital-acquired pneumonia ranges from 5 to 20 cases per 1,000 hospitalized patients. [4]. Conversely, pooled estimates from Ethiopia indicate a higher burden, with Gram-negative bacterial groups being the predominant pathogens at 37.17% [5].

In the United States, ventilator-associated pneumonia incidence ranges from 2 to 16 episodes per 1,000 ventilator-days, with most cases occurring in intensive care units (ICUs) [6]. In low-income Asian countries, pooled estimates show incidence of 18.5 episodes per 1,000 ventilator-days [7]. A study from Nepal reported ventilator-associated pneumonia incidence of 20%, while an Ethiopian assessment identified an even higher cumulative incidence of 27.9% and a rate of 45.7 episodes per 1,000 ventilator-days [8].

The aetiology of hospital-acquired pneumonia and ventilator-associated pneumonia is well recognized to be polymicrobial in nature. A wide spectrum of bacterial pathogens has been implicated, including *Acinetobacter* spp., *Chryseobacterium* spp., *Enterobacter* spp., *Elizabethkingia meningoseptica*, members of the *Enterobacteriaceae* family such as *Escherichia coli*, *Klebsiella* spp., and *Proteus* spp., as well as *Pseudomonas aeruginosa*, *Serratia marcescens*, *Staphylococcus aureus* (both methicillin-sensitive [MSSA] and methicillin-resistant [MRSA] strains), *Stenotrophomonas maltophilia*, *Streptococcus pneumoniae*, and other *Streptococcus* species [9–11].

*Pseudomonas aeruginosa* is among the top five major etiologic agents of both hospital-acquired pneumonia and ventilator-associated pneumonia, often correlating with poor clinical outcomes and extensive antimicrobial resistance [12–14]. Responsible for roughly 7% of all healthcare-associated infections [1], *P. aeruginosa* is a key member of the ESKAPE group of pathogens (*Enterococcus faecium*, *Staphylococcus aureus, Klebsiella pneumoniae, Acinetobacter baumannii, P. aeruginosa, and Enterobacter* spp.). These pathogens are considered highly virulent, possess significant antibiotic resistant properties, and collectively account for a substantial proportion of healthcare-associated infections [15,16]. The World Health Organization has further designated *P. aeruginosa* as a priority bacterial pathogen for 2024 due to its significant public health burden, its critical role in antimicrobial resistance, and its involvement in severe clinical infections [17].

In the US, *P. aeruginosa* showed a 26.5% and 27.1% resistance to cephalosporins and fluoroquinolones, respectively [18]. Several countries reported even higher resistance, with rates up to 60% [19]. Although recent studies indicate a declining trend in multidrug resistance [20], carbapenem-resistant strains remain a major concern for the World Health Organization because of their negative impact on patient outcomes and healthcare systems. Notably, the U.S. Centres for Disease Control and Prevention (US CDC) recorded a decrease in multidrug-resistant (MDR) *P. aeruginosa* from 16% in 2011 to 9% in 2018 [20].

By the close of 2023, Ethiopia’s hospital count exceeded 480 facilities. Notably, comprehensive specialized hospitals constituted approximately 5% of the total. Of all the hospitals, around 10% were located in the capital city, Addis Ababa including 16 public facilities [21]. Healthcare-associated infection studies conducted in Ethiopia have consistently demonstrated the highest prevalence in Addis Ababa. Therefore, the current research focuses on tertiary-level hospitals within the capital.

Understanding the factors that influence the persistence of hospital-acquired pneumonia is essential, particularly in Ethiopia, where substantial knowledge gaps driven by the absence of a formal surveillance system limit insight into the true burden and underlying determinants. Utilizing data from three major public hospitals in Ethiopia, our study aims to determine the prevalence, microbiological profiles and associated factors of hospital-acquired pneumonia. In addition, it evaluates the role of *P. aeruginosa* in hospital-acquired pneumonia and characterizes its antibiotic resistance profiles.

We anticipate that the findings of this study will provide valuable insights for practitioners and hospital administrators to strengthen Infection Prevention and Control (IPC) practices and support the implementation of effective antibiotic stewardship programs, ultimately improving the quality of patient care within healthcare facilities.

## Materials and methods

### Study design

A facility-based cross-sectional design was employed targeting patients with hospital-acquired pneumonia in hospitals in Addis Ababa between September 2022 and April 2024. Employing a single proportion formula in EPI Info version 7, we calculated a sample size of 217. Key parameters used included an expected hospital-acquired pneumonia prevalence of 16%, a 95% confidence interval, a 5% margin of error, and an adjustment of 5% for non-response. The study screened 1,800 patients with suspected hospital-acquired pneumonia; of these, 313 cases were confirmed to meet the inclusion criteria based on physicians’ diagnoses.

### Study area

The study was conducted in three public hospitals in Ethiopia’s capital city, Addis Ababa: Tikur Anbessa Specialized Hospital (TASH), St. Paul’s Hospital Millennium Medical College (St. Paul’s), and St. Peter’s Specialised Hospital (St. Peter’s). Selection was based on their designation as referral centres handling critically ill patients from throughout the country. These characteristics were hypothesized to elevate the risk of hospital-acquired pneumonia.

### Data collection and procedure

Patients with hospital-acquired pneumonia, confirmed by physicians according to the US CDC criteria integrating mainly clinical and radiological evidence [22], were subsequently included in the study population. The inclusion criteria are clinical and radiological confirmed pneumonia patients admitted for at least 48 hours and aged over 18 years. Prior to data collection, study participants were provided with and signed an IRB-approved standard consent form in two local languages, indicating their informed consent. Data were collected via a structured instrument covering demographic characteristics and hospitalization history, augmented by a review of patient records for additional data on current admission conditions, diagnostic procedures, complications, and treatments. Upon completion of the interview, early morning sputum or tracheal aspirates were collected from patients using sterile containers, placed in cooler boxes and transported to the microbiology laboratory at the Ethiopian Public Health Institute within a two-hour window [23–24].

Following a 24-hour incubation on Cetrimide agar, the colonies grown from the respiratory samples were identified using oxidase biochemical testing and subsequently processed for further identification by Matrix-Assisted Laser Desorption Ionization–Time of Flight (MALDI-TOF) mass spectrometry at the Animal Health Institute of Ethiopia and Wudase Diagnostic Laboratory in Addis Ababa [25–26]. Of the clinical isolates positively identified as respiratory pathogens by MALDI-TOF, all were preserved and *P. aeruginosa* isolates were further subjected to antimicrobial susceptibility testing via the Kirby-Bauer disk diffusion method. Disks impregnated with amikacin, tobramycin, piperacillin-tazobactam, ceftazidime, ciprofloxacin, cefepime, and meropenem were placed on Mueller-Hinton agar plates and incubated for 18 hours at 35°C [27]. Inhibition zone diameters were then measured, and sensitivity was interpreted as susceptible, intermediately susceptible, or resistant according to the European Committee on Antimicrobial Susceptibility Testing (EUCAST) standards [28].

### Data entry and analysis

Data were entered into Epi Info^Tm^ 7. Data cleaning and management were performed, descriptive and advanced statistical analyses, including binary, logistic, and multiple regressions, were conducted using statistical package for social sciences (SPSS) version 24 and statistical software package (STATA) version 17. We excluded five variables from the analysis due to missing values exceeding 15%. We applied a list wise deletion, as they were neither critical exposures nor primary outcomes for the analysis. The excluded data include pneumonia complication types, antibiotics given and laboratory tests and the corresponding results.

### Data quality

For each batch of cultured samples, a confirmed reference strain of *P. aeruginosa* ATCC 27853 [29] standard control strains was inoculated onto cetrimide agar alongside the clinical samples. In all instances, this positive control demonstrated consistent growth, confirming the medium’s ability to support *P. aeruginosa* growth.

### Study variables

The outcome variables include the prevalence of hospital-acquired pneumonia (proportion of hospitalized patients who develop pneumonia 48 hours or more after hospital admission, among the total number of hospitalized patients screened), the prevalence of ventilator-associated pneumonia (proportion of hospitalized patients who develop pneumonia 48 hours or more after hospital admission, among the total number of hospitalized patients assessed), and the prevalence of antibiotic resistant *P. aeruginosa (*proportion of *P. aeruginosa* isolates that demonstrate resistance to one or more antimicrobial agents as defined by EUCAST). The predictor variables include age, sex, alcohol consumption, smoking status, underlying medical conditions, length of hospital stay, aspiration, and ICU admission.

### Confounders and bias

Multivariable logistic regression was used to adjust for potential confounders which include comorbidities. Regular consultations were held with physicians to ensure consistent application of inclusion criteria across the three hospitals. The cold chain was strictly maintained to preserve sample quality from the point of collection through transport and subsequent storage. Culture and antimicrobial susceptibility testing were performed with appropriate quality control measures to ensure reliability.

### Ethical clearance

This study was approved by the Institutional Review Board (IRB) of College of Health Sciences in Addis Ababa University under approval number 077/21/SPH before the commencement of data collection.

## Results

### Demographics

During the study period a total of 1,800 patients were identified as having a presumptive hospital-acquired pneumonia in the three hospitals. Physicians confirmed 313 of these cases as hospital-acquired pneumonia. Due to insufficient or poor-quality clinical samples, fifteen patients were excluded, resulting in a final study population of 298 patients.

Forty-one percent of the patients were female and 59% were male. Most were married (71%), while 24% were single and 4% were divorced. The mean age was 44 years; 28% were between 30–39 years old, and 9% were aged over 70 years. Fifty-eight percent had received formal education, and among them, 15% had completed at least an elementary-level education (Table 1). Regarding occupation, 45% were skilled professionals and 42% were unemployed. With respect to hospital wards, 55% were drawn from ICUs, 42% from medical departments, and 2% from emergency departments.

**Table 1:** Sociodemographic and clinical characteristics of patients with hospital-acquired pneumonia enrolled in the study at TASH, St. Paul’s and St. Peter’s hospitals, Addis Ababa, Ethiopia.

Variables	TASH (n = 50)	St. Paul’s (n = 164)	St. Peter’s (n = 84)	Total (N = 298)
Sex, n (%)				
Female	26 (52.0)	63 (38.4)	32 (38.1)	121 (40.6)
Male	24 (48.0)	101 (61.6)	52 (61.9)	177 (59.4)
Age category, n (%)				
20–29 years	14 (32.6)	34 (24.1)	4 (5.5)	52 (20.2)
30–39 years	10 (23.3)	27 (19.1)	35 (47.9)	72 (28.0)
40–49 years	6 (14.0)	16 (11.3)	17 (23.3)	39 (15.2)
50–59 years	9 (20.9)	26 (18.4)	11 (15.1)	46 (17.9)
60–69 years	3 (7.0)	20 (14.2)	3 (4.1)	26 (10.1)
≥70 years	1 (2.3)	18 (12.8)	3 (4.1)	22 (8.6)
Marital status, n (%)				
Single	18 (36.0)	44 (26.8)	11 (13.1)	73 (24.5)
Married	30 (60.0)	118 (72.0)	64 (76.2)	212 (71.1)
Divorced/Separated	2 (4.0)	2 (1.2)	9 (10.7)	13 (4.4)
Education level, n (%)				
Read & write	10 (25.6)	13 (12.4)	38 (47.5)	61 (27.2)
Elementary school	11 (28.2)	19 (18.1)	4 (5.0)	34 (15.2)
Secondary school	10 (25.6)	58 (55.2)	5 (6.3)	73 (32.6)
Graduate	8 (20.5)	15 (14.3)	28 (35.0)	51 (22.8)
Post-graduate	0 (0.0)	0 (0.0)	5 (6.3)	5 (2.2)
Admission ward, n (%)				
Adult ICU	1 (2.0)	162 (98.8)	0 (0.0)	163 (54.7)
Emergency	8 (16.0)	0 (0.0)	2 (2.4)	10 (3.4)
Medical	41 (82.0)	2 (1.2)	82 (97.6)	125 (41.9)
Past hospitalization, n (%)				
Yes	19 (38.0)	147 (89.6)	14 (16.7)	180 (60.4)
No	31 (62.0)	17 (10.4)	70 (83.3)	118 (39.6)

Sixty-one percent of the patients had a history of hospitalization within the past three months with a chief compliant of shortness of breath in 9% of cases. Among the study subjects, 7% required mechanical ventilation support. The average hospital stay was 13 days. Approximately 20% of the patients developed a hospital-acquired pneumonia, and 67% were primarily treated with cephalosporin. Additionally, 85% received antibiotics for at least 7 days.

Respiratory diseases (30%), infectious diseases (29%), and neurological disorders (11%) were the top three reasons for hospitalization as described in Table 2 below. Nearly 70% of the patients were admitted as emergency cases. The length of hospital stay ranged from 3 to 65 days, with an average of 8.29 days.

**Table 2:** Major reasons for hospital admission among patients with hospital-acquired pneumonia at TASH, St. Paul’s and St Peter’s hospitals, Addis Ababa, Ethiopia.

Primary admission reasons	TASH (n = 46)	St. Paul’s (n = 159)	St. Peter’s (n = 76)	Total (N = 281)
Respiratory diseases	7 (15.2)	65 (40.9)	12 (15.8)	84 (29.9)
Infectious diseases	5 (10.9)	18 (11.3)	59 (77.6)	82 (29.2)
Neurological disorders	9 (19.6)	23 (14.5)	0 (0.0)	32 (11.4)
Cardiovascular diseases	6 (13.0)	14 (8.8)	2 (2.6)	22 (7.8)
Renal and urinary diseases	2 (4.3)	15 (9.4)	2 (2.6)	19 (6.8)
Gastrointestinal and hepatic diseases	2 (4.3)	9 (5.7)	0 (0.0)	11 (3.9)
Autoimmune and hematologic disorders	8 (17.4)	2 (1.3)	0 (0.0)	10 (3.6)
Endocrine and metabolic disorders	2 (4.3)	7 (4.4)	1 (1.3)	10 (3.6)
Miscellaneous conditions	2 (4.3)	6 (3.8)	0 (0.0)	8 (2.8)
Oncological disorders	3 (6.5)	0 (0.0)	0 (0.0)	3 (1.1)
Total	46 (100.0)	159 (100.0)	76 (100.0)	281 (100.0)

### Prevalence

**Fig 1:**
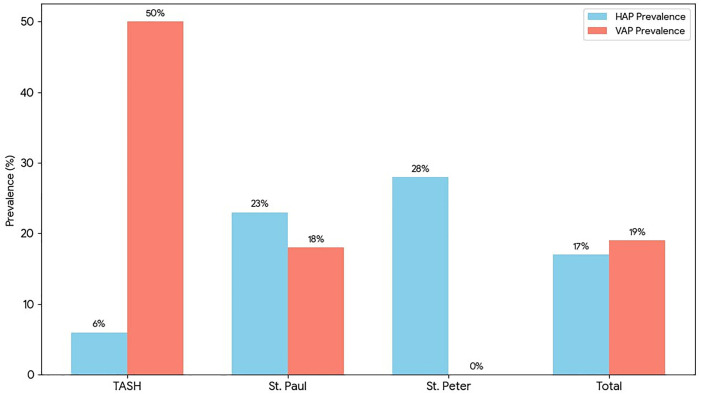
Prevalence of hospital-acquired and ventilator-associated pneumonia among patients at TASH,St. Peter’s andSt. Paul’s hospitals, Addis Ababa, Ethiopia.

We detected 17% prevalence of hospital-acquired pneumonia across the three hospitals (Fig 1). Among hospital-acquired pneumonia cases, 55% were from ICUs, and 41% were from medical departments. Ventilator-associated pneumonia accounted for 19% of cases, the majority of which occurred in ICU of St. Paul’s hospital.

**Fig 2:**
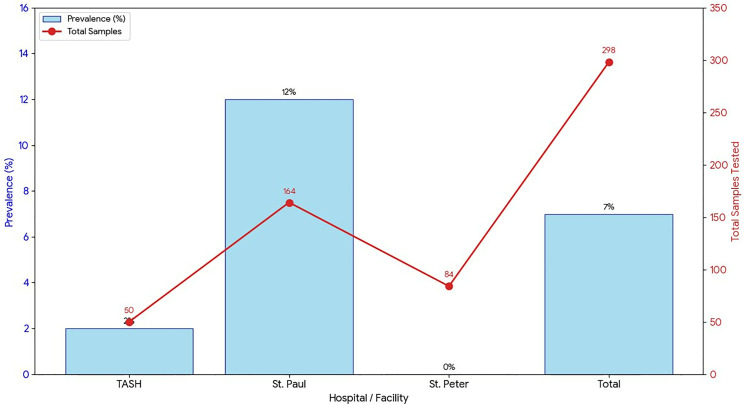
Prevalence of *Pseudomonas aeruginosa* among hospital-acquired pneumonia cases at TASH, St. Peter’s and St. Paul’s hospitals, Addis Ababa, Ethiopia.

We detected a 7% prevalence of *P. aeruginosa* in the respiratory samples across the three hospitals (Fig 2). St. Paul exhibited the highest burden with 12% prevalence, whereas TASH reported a low prevalence of 2%, and St. Peter’s recorded no positive samples.

The term ESKAPE, introduced by Louis B. Rice in 2008, refers to a group of highly drug-resistant bacteria that cause major healthcare-associated infections by “escaping” many antibiotics [30]. In our study, 28% of patients tested positive for ESKAPE pathogens, as shown in Table 3.

**Table 3:** Percentage of ESKAPE pathogens identified from respiratory samples of patients with hospital-acquired pneumonia at TASH, St. Paul’s and St Peter’s hospitals, Addis Ababa, Ethiopia.

ESKAPE pathogen	TASH (n)	St. Paul’s (n)	St. Peter’s (n)	Total (n)
*Enterococcus faecium*	1	0	0	1
*Staphylococcus aureus*	4	5	4	13
*Klebsiella pneumoniae*	1	4	1	6
*Acinetobacter baumannii*	3	17	4	24
*Pseudomonas aeruginosa*	1	20	0	21
*Enterobacter* spp.	0	1	0	1
Total	10	42	9	61

### Presence of potential human pneumonia pathogens

Among the 298 respiratory samples analysed, 89 (30%) yielded pneumonia-causing bacteria (Table 4). *Acinetobacter baumannii* was the most frequently detected pathogen, accounting for 8% of all samples, followed by *Staphylococcus aureus* (4%), *Stenotrophomonas maltophilia* (3%), and *Pseudomonas aeruginosa* (2%). Other identified pathogens include *Klebsiella pneumoniae*, *Klebsiella aerogenes*, *Citrobacter freundii*, *Elizabethkingia anophelis*, *Rothia* species, *Serratia marcescens*, and several rare bacteria were detected at low frequencies.

**Table 4:** List and number of pneumonia causing pathogens identified from respiratory samples of patients with hospital-acquired pneumonia at TASH, St. Paul’s and St Peter’s hospitals, Addis Ababa.

Bacterial species	TASH (n = 6)	St. Paul’s (n = 48)	St. Peter’s (n = 7)	Total (n = 92)	Percentage (%)
*Acinetobacter baumannii*	3	17	4	24	26.1
*Pseudomonas aeruginosa*	1	20	0	21	22.8
*Staphylococcus aureus*	4	5	4	13	14.1
*Stenotrophomonas maltophilia*	0	9	2	11	12.0
*Klebsiella pneumoniae*	1	5	0	6	6.5
*Rothia mucilaginosa*	0	3	0	3	3.3
*Citrobacter freundii*	0	2	0	2	2.2
*Elizabethkingia anophelis*	0	2	0	2	2.2
*Achromobacter xylosoxidans*	1	0	0	1	1.1
*Enterococcus faecalis*	1	0	0	1	1.1
*Escherichia coli*	0	0	1	1	1.1
*Legionella longbeachae*	0	0	1	1	1.1
*Pandoraea pnomenusa*	0	1	0	1	1.1
*Proteus mirabilis*	0	1	0	1	1.1
*Providencia rettgeri*	0	1	0	1	1.1
*Rothia aeria*	0	1	0	1	1.1
*Rothia dentocariosa*	0	1	0	1	1.1
*Serratia marcescens*	0	1	0	1	1.1
Total isolates	11	69	12	92	100.0

### Resistance patterns of *P. aeruginosa* isolates

Patients positive for *P. aeruginosa* were further subjected to antimicrobial susceptibility, and the results are presented in Table 5. Overall, 19 isolates were processed for antimicrobial susceptibility testing against seven anti-*Pseudomonas* drugs as per the national standards.

**Table 5:** Antimicrobial sensitivity and resistance percentages of *P. aeruginosa* isolated from respiratory samples of patients with hospital-acquired pneumonia at TASH and St. Paul’s hospitals, Addis Ababa, Ethiopia.

Antimicrobial agents	TASH		St. Paul		Total	
	S	R	S	R	S	R
Amikacin (30 µg)	0	0	90%(n = 9)	10%(n = 1)	90%(n = 9)	10%(n = 1)
Tobramycine (10 µg)	2%(n = 1)	0	3%(n = 4)	8%(n = 13)	28%(n = 5)	72%(n = 13)
Ciprofloxacin (5 µg)	2%(n = 1)	0	5%(n = 8)	6%(n = 10)	47%(n = 9)	53%(n = 10)
Ceftazidime (30 µg)	0	2%(n = 1)	1%(n = 2)	10%(n = 15)	11% (n = 2)	89% (n = 16)
Cefepime (30 µg)	0	2%(n = 1)	1%(n = 2)	10%(n = 16)	11%(n = 2)	89% (n = 17)
Piperacillin-tazobactam (100/10 µg)	2%(n = 1)	0	8%(n = 12)	2%(n = 3)	81% (n = 13)	19% (n = 3)
Meropenem (10 µg)	0	2% (n = 1)	6% (n = 10)	5%(n = 8)	53% (n = 10)	47% (n = 9)

S=Susceptible, R=Resistant.

The results reveal elevated resistance to third- and fourth-generation cephalosporins (89%), tobramycin (72%), and ciprofloxacin (53%). Nearly half of the isolates were resistant to meropenem, indicating presence of carbapenem-resistant *P. aeruginosa*. In contrast, amikacin showed the highest activity, with 90% susceptibility, followed by piperacillin–tazobactam with 81% susceptibility. Overall, isolates from TASH demonstrated lower susceptibility compared to those from St. Paul, highlighting significant variability between facilities.

Out of 18 total isolates, 16 (88.9%) exhibited some level of resistance, including 4 with single-drug resistance and 12 classified as MDR. The majority of MDR cases (12/18, 66.7%) were concentrated in the St. Paul Adult ICU, which accounted for 17 of the 18 total isolates and all MDR isolates (Table 6). In contrast, TASH reported only a single non-MDR resistant isolate (Table 4).

**Table 6:** Multi-drug resistance profiles of *P. aeruginosa* isolated from respiratory samples of patients with hospital-acquired pneumonia at TASH and St. Paul’s hospitals, Addis Ababa, Ethiopia.

Hospital Name	Ward Name	Number of susceptible isolates	Number of resistance isolates	MDR	Total
St. Paul	Adult ICU	2	3	12	17
TASH	Medical	0	1	0	1
Total		2	4	12	18

MDR = Multidrug resistant.

### Factors associated with the presence of pneumonia-causing pathogens

**Fig 3:**
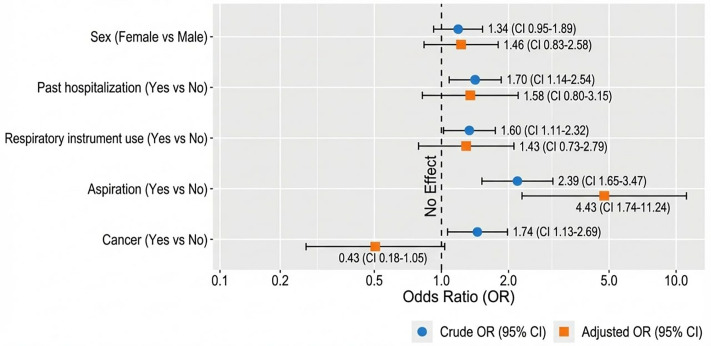
Odds ratios linking patient and hospital factors to pneumonia pathogens in patients with hospital-acquired pneumonia at TASH, St. Paul’s and St. Peter’s hospitals, Addis Ababa, Ethiopia.

In our chi-square analysis, past hospitalization, use of respiratory instruments, aspiration, and cancer were significantly associated with the presence of pneumonia-causing pathogens, whereas sex was not (Fig 3). In multivariable logistic regression analysis, only aspiration remained independently associated with pneumonia pathogen presence, with patients experiencing aspiration having more than fourfold increased odds of pneumonia-causing pathogens (AOR = 4.43, 95% CI: 1.74–11.24, p = 0.002).

## Discussion

A growing body of evidence highlighting the role of antimicrobial resistance in healthcare-associated infections underscores the substantial challenges faced by healthcare systems in low-income countries in safeguarding patients from adverse events during hospitalization.

This study was conducted in a context where surveillance systems for healthcare-associated infections are lacking, access to appropriate and affordable diagnostic technologies is limited, and infection prevention and control practices remain suboptimal. In this setting, the generation of facility-level evidence on the microbiological profiles of causative agents and antimicrobial resistance trends for prevalent conditions such as hospital-acquired pneumonia and key pathogens like *P. aeruginosa* will support clinicians in improving the management of healthcare-associated infections and contribute to reducing preventable mortality [31].

### Prevalence

Our study identified a 17% (n = 298) prevalence of hospital-acquired pneumonia across three hospitals. This estimate is lower than the 24.7% reported at Zewditu Memorial Hospital by Tassew et al. [32], but higher than the 13.5% reported at AaBET Hospital by Yemene [33], both in Addis Ababa. These differences likely reflect methodological and setting-related factors. The lower prevalence at AaBET may be due to reliance on culture-confirmed cases, which can underestimate pneumonia because of diagnostic limitations, while the higher prevalence at Zewditu may be influenced by its prospective design and broader clinical case ascertainment. Our intermediate prevalence likely reflects the focus on referral hospitals and intensive care settings, where patients are more critically ill and exposed to invasive procedures, as well as a longer study period with systematic screening.

Our study found that ventilator-associated pneumonia accounted for 19% of hospital-acquired pneumonia cases. This proportion is lower than the 31.3% prevalence reported by Tegegne et al. among intubated adult ICU patients in public hospitals in Addis Ababa [34], likely due to their exclusive focus on mechanically ventilated patients and high-risk exposures such as re-intubation, prolonged ventilation, and tracheostomy. Similarly, a study from Pakistan by Nisar et al. reported a higher prevalence (25.6%) among ventilated ICU patients [35], which may reflect its ICU-only design, longer ventilation durations, and use of combined clinical, radiological, and microbiological diagnostic criteria. Despite these differences, all studies consistently indicate that ventilator-associated pneumonia remains a common complication among mechanically ventilated patients. The lower prevalence observed in our study likely reflects an inclusion of a broader hospital population rather than a lower intrinsic risk of ventilator-associated pneumonia cases.

In our study, non-fermenting Gram-negative bacteria, particularly *Acinetobacter baumannii* (26%) and *Pseudomonas aeruginosa* (23%), were the predominant pathogens, whereas *Staphylococcus aureus* (14%) and *Klebsiella pneumoniae* (7%) were less frequent. Compared to South Africa [36] and Romania [37], where *S. aureus* and *K. pneumoniae* were more common, our data highlight a distinct local burden of non-fermenting Gram-negative pathogens in hospital-acquired infections. These findings underscore the importance of facility-level surveillance and targeted infection prevention to address the unique pathogen distribution in Ethiopian hospitals.

In our study, *P. aeruginosa* accounted for 7% of all screened hospital-acquired pneumonia patients, indicating a meaningful contribution within a mixed ward population across three referral hospitals. This prevalence is lower than that reported in a multinational ICU cohort study, where *P. aeruginosa* accounted for 18.4% of nosocomial lower respiratory tract infections [38], and also lower than the Chinese meta-analysis, which reported a prevalence of 17.8% among hospital-acquired pneumonia cases [39]. In contrast, a Pakistani ICU-based study reported a substantially higher prevalence, with *P. aeruginosa* accounting for 30.6% of hospital-acquired pneumonia cases [40]. This marked difference likely reflects the study’s restriction to a single medical ICU and smaller sample size. Overall, our findings fall at the lower end of internationally reported *P. aeruginosa* prevalence. ICU-focused studies typically report rates of 18–20% or higher, with variation largely reflecting differences in patient acuity, ICU exposure, diagnostic practices, and local microbial ecology.

### Resistance profile

Our findings indicate that none of the eight drugs used for treatment of *Pseudomonas* infections were fully potent. Amikacin was found to have the lowest resistance level at 10%, which is similar to a Chinese study that reported just a little higher 22% resistance rate for amikacin [39], accentuating the potential of this drug as a viable option for management of cases.

We detected resistance levels of approximately 89% to cephalosporin (Cefepime and Ceftazidime) and 45% resistance to carbapenems. These rates are significantly higher than those reported in a multi-year study conducted by AlBahrani et al. in Saudi Arabia, which indicated an overall resistance of just 9% for both cephalosporin and carbapenems [41]. The diminishing efficacy of these drugs rings a bell in terms of the alarming resistance rate creeping in the health care systems in resource-constrained settings.

Sixty-seven percent of the isolates in our study exhibited MDR, which is significantly higher than the 15–30% range reported in the scientific literature [42]. Among the ventilator-associated pneumonia cases, 75% were found to be infected with multidrug-resistant *P. aeruginosa*. This is even higher than the global average estimated by Li et al., to be about 33% [43]. The stark contrast is an indication of the high MDR ventilator-associated pneumonia burden in our study population that may have been attributed to local factors such as empirical treatment, poor diagnostic stewardship practices and poor infection prevention practices.

### Pathogen determinants

Our study demonstrates ICUs account for the highest proportion of hospital-acquired pneumonia and ventilator-associated pneumonia cases, placing patients receiving critical care and undergoing invasive procedures at substantial risk. In contrast, the emergency ward had the fewest cases, possibly due to shorter patient stays or a lower proportion of high-risk patients. These findings are consistent with studies from France by Bernardo Guzmán-Herrador et al. and from China, which reported ICUs as high-risk settings for hospital-acquired pneumonia and ventilator-associated pneumonia [44], but contrast with another Chinese study indicating that general wards contributed more cases than ICUs [31], suggesting that the primary hotspots for hospital-acquired pneumonia may vary according to local factors. The higher incidence of hospital-acquired pneumonia and ventilator-associated pneumonia in intensive care units is likely attributable to the critical illness of patients and the frequent use of invasive procedures [45].

In our multivariable logistic regression analysis, only aspiration remained independently associated with pneumonia pathogen presence, with patients experiencing aspiration having more than fourfold increased odds of pneumonia-causing pathogens. This finding is consistent with a Swedish study that identified aspiration as a strong risk factor for hospital-acquired pneumonia [46]. The observed association underscores the critical role of aspiration in the pathogenesis of hospital-acquired pneumonia, irrespective of the study setting.

### Strengths and limitations

This study used a combination of clinical assessment, radiological findings, and laboratory results to confirm hospital-acquired pneumonia cases, ensuring a high level of diagnostic accuracy, and employed MALDI-TOF for pathogen identification, which offers greater precision than conventional culture methods. However, the study population was not fully representative of all hospital-acquired and ventilator-associated pneumonia cases, as it was limited to selected wards (ICU, medical, surgical, and emergency) chosen for logistical reasons. Additionally, on-going antibiotic use among many patients at the time of sampling may have led to underestimation of the true prevalence, and the study did not distinguish between microbial colonization and true infection, which remains a key limitation.

## Conclusions

This study demonstrates a substantial burden of hospital-acquired pneumonia among young to middle-aged male adults with prior healthcare exposure and severe underlying respiratory disease, potentially driven predominantly by ESKAPE pathogens.

The high prevalence of cephalosporin resistance and multidrug resistance among *P. aeruginosa*, together with aspiration as a key risk factor, suggests the occurrence of highly resistant pneumonias that are often treatable only with last-line and costly antimicrobial agents. These findings indicate deficiencies in hospital defences against drug-resistant organisms and an increased risk of adverse patient outcomes.

These findings necessitate a fundamental shift in infection prevention strategies, emphasizing rigorous aspiration prevention measures and strengthened diagnostic stewardship to ensure the judicious use of antimicrobials in these hospitals. Addressing this challenge requires moving beyond generalized standard control measures toward targeted and tailored interventions. Furthermore, we propose that environmental surveillance and molecular epidemiological studies be prioritized in future research.

## Supporting information

S1 File Flow chart of clinical sample collection, transportation, and identification procedures. This is the S1 File legend. This file presents high-level technical steps and key methodological decisions related to respiratory sample collection, transportation, culturing, diagnostic testing, and antimicrobial susceptibility testing for *Pseudomonas aeruginosa*, along with selected associated images.
(TIF)

S2 File Supplementary dataset (Excel format). This is the S2 File legend. This file contains, in Excel format, the dataset for this study, comprising all collected variables, including both independent and dependent variables analyzed.
(XLSX)
